# The Immunomodulatory Effect of the Gut Microbiota in Kidney Disease

**DOI:** 10.1155/2021/5516035

**Published:** 2021-05-15

**Authors:** Mingxuan Chi, Kuai Ma, Jing Wang, Zhaolun Ding, Yunlong Li, Shaomi Zhu, Xin Liang, Qinxiu Zhang, Linjiang Song, Chi Liu

**Affiliations:** ^1^Reproductive & Women-Children Hospital, School of Medical and Life Sciences, Chengdu University of Traditional Chinese Medicine, Chengdu, China; ^2^Department of Nephrology, Osaka University Graduate School of Medicine, Osaka, Japan; ^3^Reproductive Center, Shuguang Hospital Affiliated to Shanghai University of Traditional Chinese Medicine, Shanghai, China; ^4^Department of Emergency Surgery, Shaanxi Provincial People's Hospital, Xi'an, Shaanxi, China; ^5^Department of Nephrology, Sichuan Clinical Research Center for Kidney Disease, Sichuan Provincial People's Hospital, University of Electronic Science and Technology, Chengdu, China; ^6^Chinese Academy of Sciences Sichuan Translational Medicine Research Hospital, Chengdu, China

## Abstract

The human gut microbiota is a complex cluster composed of 100 trillion microorganisms, which holds a symbiotic relationship with the host under normal circumstances. Intestinal flora can facilitate the treatment of human metabolic dysfunctions and interact with the intestinal tract, which could influence intestinal tolerance, immunity, and sensitivity to inflammation. In recent years, significant interests have evolved on the association of intestinal microbiota and kidney diseases within the academic circle. Abnormal changes in intestinal microbiota, known as dysbiosis, can affect the integrity of the intestinal barrier, resulting in the bacterial translocation, production, and accumulation of dysbiotic gut-derived metabolites, such as urea, indoxyl sulfate (IS), and p-cresyl sulfate (PCS). These processes lead to the abnormal activation of immune cells; overproduction of antibodies, immune complexes, and inflammatory factors; and inflammatory cell infiltration that can directly or indirectly cause damage to the renal parenchyma. The aim of this review is to summarize the role of intestinal flora in the development and progression of several renal diseases, such as lupus nephritis, chronic kidney disease, diabetic nephropathy, and renal ischemia-reperfusion injury. Further research on these mechanisms should provide insights into the therapeutic potential of regulating intestinal flora and intervening related molecular targets for the abovementioned nephropathy.

## 1. Introduction

Kidney disease is a general term for renal heterogeneous disorders affecting the kidney structure and function, which is a dominant contributor to global morbidity and mortality [1]. Although it has been increasingly identified as a significant public health problem worldwide with increasing prevalence and poor outcomes, clinical diagnosis and therapeutic interventions are lagging. Nowadays, most therapeutic methods are limited to lowering blood pressure, controlling blood glucose, and reducing proteinuria [2]. Current studies are aimed at developing more effective therapeutic strategies to prevent the progression of renal diseases. Over the past years, our understanding of the composition and function of the gut microbiota has been expanded, mainly on account of the evolution and advances of modern molecular techniques. Developing research studies on gut microbiota have shown that this formerly “neglected organ” plays a significant role in many diseases within and beyond the intestinal tract. Substantial differences of the gut microbiota composition, immunogenicity, and metabolic activity have been observed by comparing healthy individuals to patients presenting with different types of kidney diseases [3–6] and other noncommunicable illnesses, such as diabetes mellitus, obesity, atherosclerotic cardiovascular disease, heart failure, and liver diseases [7–11]. These changes of the gut microbiota are causally correlated with disease phenotypes, complications, and outcomes according to experimental studies in animals and humans [7, 12, 13].

The human gut microbiota, also known as the intestinal flora, is composed of ~100 trillion microorganisms constituted by a broad spectrum of over 500 genera of bacteria from two main phyla, namely, *Bacteroidetes* and *Firmicutes*. Generally, the diversity and abundance of the intestinal microbiota differ along the intestinal tract and maintain a dynamic balance. Known as the body's “second brain,” the intestinal microbiota plays a major role in the absorption and metabolism of nutrients, hormone secretion, and toxin degradation, which enable it to control the human intestinal homeostasis and even the whole internal environment. The SCFAs including acetate, propionate, and butyrate are predominant final products of the distal gut microbiome, fermented from a variety of plant polysaccharides produced under anaerobic conditions [14]. SCFAs can provide about 10% of the caloric needs for the human body [15] and enhance the barrier function by regulating the retinol production and mediating the secretion of mucin and IgA [16]. In addition, SCFAs come into the blood circulation and then exert their systemic effects such as increasing anti-inflammatory factors, downregulating autoimmunity-related factors, and developing regulatory T (Treg) cells [17–20] via the G protein-coupled receptors (e.g., GPR41, GPR43, and GPR109A). Furthermore, accumulating pieces of evidence have reported the positive effects of SCFAs in treating kidney problems caused by several diseases [13, 21–24].

**Figure 1:**
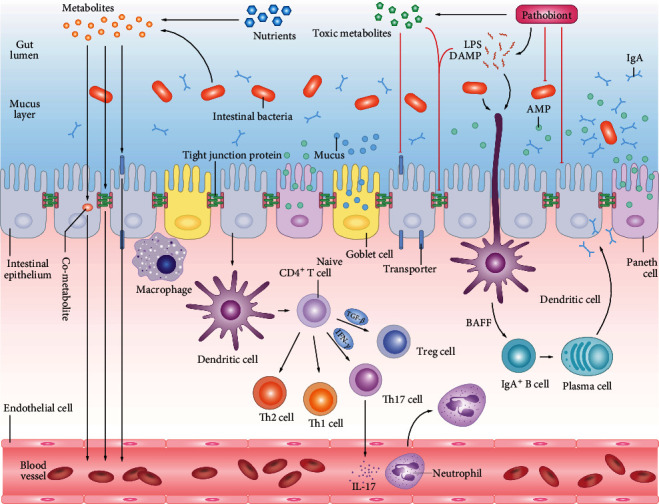
The intestinal epithelial barrier allows a large number of metabolites and immune signals to pass in both directions while blocking the pathways of pathogenic bacteria, toxic metabolites, and microbial byproducts. The outermost layer of the intestinal barrier is the mucus layer which is composed of mucin glycoprotein, AMPs, and sIgA, produced by goblet cells, Paneth cells, and plasma cells, respectively, and excludes the microbiome from the epithelial surface. Adjacent cells are linked together by the tight junction protein families that can determine the permeability and prevent mechanical disruption of the epithelial sheet. The intestinal immune system is established and mature with the participation of the gut microbiota. Once bound to luminal antigens, DC pattern recognition receptors express costimulatory molecules and cellular factors involved in regulating Th1, Th2, Th17, and Treg cells differentiated from naive CD4^+^ T cells, maintaining Treg/Th17 balance and forming immune homeostasis. The intestinal microbiome promotes the differentiation of IgA-secreting plasma cells by activating APRIL (a proliferation-inducing ligand) receptor and B cell-activating factor in DC.

In addition to the metabolic function, the gut microbiota performs some basic roles to promote the maturation of intestinal immunity [25–28] and maintain the integrity of the intestinal epithelial barrier to prevent the invasion and colonization of pathogenic microorganisms [29]. The intestinal epithelial barrier is essential for intestinal homeostasis, which enables the bilateral passage of vast metabolites and immune signals and simultaneously obstructs the passage of the pathogenic bacteria, toxic metabolites, and microbial byproducts [30–32]. To counterpoise these apparently contradictory roles [33, 34], the epithelial cells and immune cells closely interact with each other, establishing the first line of protection against invading pathogens. Its mechanism of action is the recruitment of phagocytes or direct bacterial prevention and killing by releasing chemokines, cytokines, AMPs, and other soluble molecules [35–38]. The intestinal immune system is built up and matures with the participation of the gut microbiota. Microfold cells (M cells) in the epithelium capture lumen contents and deliver them to the underlying antigen-presenting cells, such as macrophages and dendritic cells (DCs) [39]. Once the pattern recognition receptors of the DCs bind to the pathogenic microorganisms, the stimulated DCs that process and present the antigens express costimulatory molecules and cellular factors. These processes contribute to the regulating helper T (Th) cells, such as Th1, Th2, and Th17, and immunosuppressive Treg cells differentiated from naive CD4^+^ T cells, maintaining the Treg/Th17 balance and the immune homeostasis [14, 40–42] (Figure 1). Intestinal flora imbalance induces the activation of immune cells through this pathway, secreting a large amount of proinflammatory factors (e.g., IL-4, IL-5, IL-6, and interferon-*γ* (IFN-*γ*)), which results in immune dysregulation and inflammation. The intestinal microbiome promotes the differentiation of IgA-secreting plasma cells by activating a proliferation-inducing ligand (APRIL) receptor and B cell-activating factor (BAFF) in DCs. sIgA has a regulatory effect on intestinal microorganisms. Bacterial metabolites, such as SCFAs, histamine, spermine, and taurine, can also influence the host's immune homeostasis [43].

As a contributing factor and indicator of human health, the gut microbiota plays an important role in the prevention, diagnosis, and treatment of many human diseases. Although a dynamic balance is established between intestinal flora, host, and external environment, it is susceptible to changes caused by age, diet, antibacterial drugs, psychological pressure, and other factors, resulting in an imbalance of intestinal flora [44, 45]. Once this microecological balance of intestinal flora is destroyed, known as dysbiosis, it will lead to a variety of gastrointestinal and systemic diseases (Figure 1). Microbial dysbiosis promotes the production of bacteria-produced uremic toxins, such as IS, p-cresyl sulfate (PCS), and trimethylamine N-oxide (TMAO). These metabolites translocate into the circulation through the impaired intestinal barrier, and most of which are excreted by the kidneys, where their retention would lead to kidney dysfunction [46]. In addition to dysbiosis, the increased permeability and structural damage of the intestinal barrier result in the translocation of pathogenic bacteria and their byproducts which is a vital step leading to local or systemic inflammation [47–50], affecting various organs, including the kidneys [51, 52].

Taken together, the colonization of intestinal microorganisms is a double-edged sword for the host. The healthy microbial community plays an indispensable role in the host's nutrient absorption and metabolism, the maturation of intestinal immunity, the maintenance of the integrity of the intestinal epithelial barrier, and the prevention of colonization by pathogenic microorganisms. These are what the microbiome has contributed to the overall health of the host. However, changes in the intestinal flora can cause diseases of different organs and exacerbate existing diseases. This review summarizes the current understanding of the role of intestinal flora in the occurrence and development of kidney disease, focusing on select components of the immune system that have been shown to drive the pathogenesis of each kidney disease. Further research on the association between the immune system and the gut microbiota may contribute to the understanding of the intricate pathogenesis of kidney disease. Likewise, the regulation of intestinal flora and the intervention of related molecular targets may have a potential therapeutic utility in the treatment of kidney diseases.

## 2. Gut Microbiota in Lupus Nephritis

Systemic lupus erythematosus (SLE) is a multisystemic autoimmune disease characterized by lymphocyte overactivation and the production of antinuclear autoantibodies that drive arthritis, glomerulonephritis, and other different inflammatory tissue damage [53]. Approximately 60% of SLE patients are suffering from lupus nephritis (LN), which is one of the leading causes of morbidity and mortality in SLE, resulting in acute or chronic kidney damage through inflammation, deposition of immune complexes, and glomerular or interstitial scarring [54]. Nowadays, the etiological understanding of LN is limited in the genes and environment [55], but the specific causes still remain unclear. In recent years, the alterations of the gut microbiota have been associated with multitudinous autoimmune disorders, and present data has reported the distinctive microbiota composition in the gastrointestinal tract of LN patients [3, 56–61]. Thus, the role of intestinal flora in LN has increasingly attracted the attention of researchers [62].

In the symbiotic condition, intestinal microorganisms can affect gut tolerance, immunity, and sensitivity to inflammation through B cell maturation, Treg/Th17 ratio balance, and anti-inflammatory cytokine secretion. However, the intestinal inflammatory microenvironment in SLE patients may influence intestinal tolerance, exceeding the immunologic reactions, autoimmunity, and damage of tissues/organs in SLE patients. In the pathological circumstances of SLE, chronic inflammation disrupts the intestinal barrier, which is termed as leaky gut [63], and bacterial pathogens are directly exposed to various organs and immune systems of the body. Through the toll-like receptor (TLRs) [64, 65], antigen-presenting cells (APCs, e.g., macrophages and DCs) secrete cytokines to activate the differentiation and proliferation of T cells [14, 40]. Proinflammatory factors, such as IL-6 and IFN-*α*, are released, which play an important role in inducing B cells to release autoantibodies and cause an imbalance in the Treg/Th17 ratio [66]. A large number of autoantibodies, most of which are anticellular antibodies and antinuclear antibodies [67–69], combine with ligands to form immune complexes. Moreover, molecular mimicry may be an important link between intestinal microbiota and SLE. Bacteria can express orthologs of human Ro60 autoantigens in the SLE patients' gut. This characteristic would lead to T cell cross-reaction and the production of human anti-Ro60 autoantibodies in SLE patients [70, 71]. Particularly, it was found that *Ruminococcus gnavus* cross-reacts with human DNA, whose relative abundance in the intestinal tract is positively correlated with SLE activity and LN [72].

**Figure 2:**
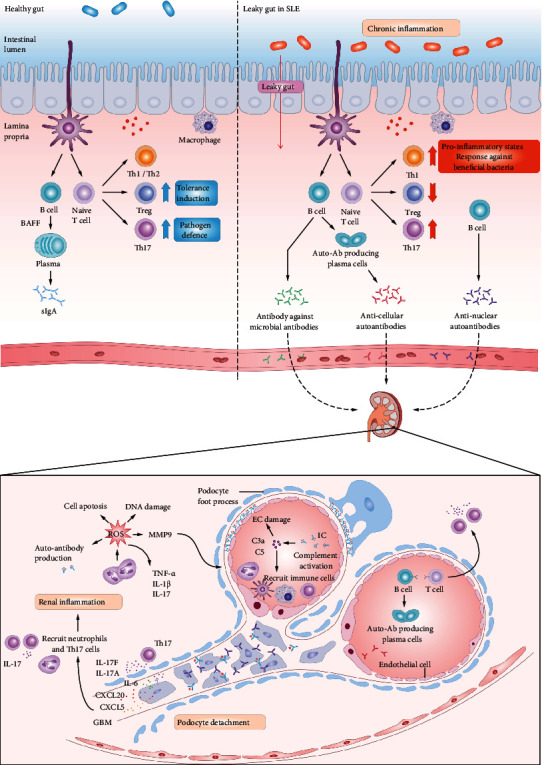
Correlation between intestinal microbiota and the incidence of lupus nephritis. Under the symbiotic condition, intestinal microorganisms can affect gut tolerance, immunity, and sensitivity to inflammation through B cell maturation, Treg/Th17 ratio balance, and anti-inflammatory cytokine secretion. In SLE patients, the intestinal inflammatory atmosphere can induce B cells to release autoantibodies, resulting in an imbalance of the Treg/Th17 ratio, leading to intestinal tolerance disorders, beyond immune response and autoimmunity, and tissue/organ damage (such as lupus arthritis (LN)). A large number of autoantibodies and immune complexes are produced and enter the circulation. The deposition of autoantibodies and immune complexes in the glomeruli leads to the activation of complement components (e.g., C3a and C5), resulting in the endothelial cell or podocyte injury and recruitment of immune cells. Infiltrated Th17 cells in the kidney secrete cytokines IL-17A and IL-17F, which activate mesangial cells and tubular epithelial cells to produce CXCL5 and CCL20, then recruit more Th17 cells and neutrophils through CCR6 and CXCR2, respectively. At the same time, ROS produced by infiltration of immune cells can lead to further renal inflammation and tissue destruction.

These changes initiated by the gut microbiota can lead to an acceleration of the process of kidney injury. The deposition of autoantibodies and immune complexes in the glomeruli leads to the activation of complement components, resulting in the injury of endothelial cells or podocytes and the recruitment of immune cells. Some pieces of evidence have reported the essential role of renal resident cells (e.g., podocytes, renal tubular epithelial cells, and glomerular mesangial cells) in the development of LN [73]. Infiltrated Th17 cells in the kidney secrete cytokines IL-17A and IL-17F, which activate the mesangial cells and tubular epithelial cells to produce C-X-C motif chemokine 5 (CXCL5) and chemokine (C-C motif) ligand 20 (CCL20) and then recruit more Th17 and neutrophils through chemokine receptor 6 (CCR6) and CXCR2, respectively. At the same time, reactive oxygen species (ROS) produced by the infiltration of immune cells can lead to further renal inflammation and tissue destruction (Figure 2).

The current treatment for LN includes the administration of high doses of corticosteroids and broad-spectrum immunosuppressants, but treatments are not ideal at present [74]. The study of the interaction between intestinal microflora and LN provides a new idea for the treatment of LN. In experimental studies, the restoration of the composition of intestinal flora through the administration of acidic water [67], vitamin A [61], probiotics [3, 75, 76], or prebiotics can moderate the inflammatory status and possibly favor renal protection in SLE models. Rodgers et al. demonstrated the renal protective potential of drug-like analogs of ES-62, which is a type of phosphorylated cholinergic glycoprotein secreted by *Acanthocheilonema viteae* and involved in maintaining the balance of regulatory/effector B cells and desensitized renal effector function [77]. Since limited reports have investigated the impact of the abovementioned treatments in SLE and LN patients, further research is needed to confirm their efficacy in clinical application.

## 3. Gut Microbiota in Chronic Kidney Disease

Chronic kidney disease (CKD) is a global health issue and is increasingly considered a social burden. More than 10% of the population has been diagnosed with CKD, in which 50% are classified as high-risk subgroups [78]. As a result of progressive renal parenchymal injury, clinical symptoms, such as the reduced glomerular filtration rate, increased urinary protein excretion, reduced synthesis of erythropoietin, and hypertension, can be noticed in patients with CKD.

CKD induces numerous alterations in internal and external factors that potentially alter the microbiota composition. Furthermore, intestinal dysbiosis is closely associated with gut inflammation and intestinal barrier disruption [79, 80]. For instance, dietary changes in CKD patients might contribute to intestinal dysbiosis and the generation of excessive uremic toxins. Urea is produced from amino acids in the urea cycle and is excreted by the kidneys (80%) and the digestive tract (20%). As renal function is impaired in patients with CKD, the digestive tract becomes the main route for urea excretion. Urea in the intestinal lumen could be converted by bacteria to NH_3_ or NH_4_OH, wherein the formation of which and increased intestinal lumen pH can promote the proliferation of pathogenic microorganisms and destroy the intestinal barrier. Other causes that probably contribute to intestinal barrier disruption in CKD include the use of numerous medications and hypervolemia [81, 82] which lead to uremia, azotemia sympathetic overactivity [83, 84], and intestinal congestion [5]. These processes lead to systemic inflammatory responses through increasing the production of proinflammatory cytokines, activating the nuclear factor-kappa B (NF-*κ*B) pathway, and dysregulating the immune response, thus exacerbating the ecological imbalance [79, 85]. The destruction of the intestinal barrier facilitates the bacterial endotoxin to enter the circulatory system, which is known as endotoxin translocation. Endotoxemia has various effects on systemic inflammation, oxidative stress, cardiac injury, and atherosclerosis [52]. More importantly, endotoxemia is positively correlated with the reduced survival of CKD and hemodialysis patients [52]. The inflammation in CKD involves the endotoxin-induced overactivation of APCs and lymphocytes [86]. However, evidence suggested that host defense against infectious microorganisms is impaired in end-stage renal disease (ESRD) patients [87, 88]. This seemingly paradoxical immune response can be explained by the endotoxin tolerance; that is, persistent innate immune activation induces immune paralysis [89], which contributes to the presence of acquired immunosuppression and systemic inflammation.

In the liver and colon, dysbiotic gut-derived uremic toxins, such as indoles and phenols, are further metabolized into TMAO, IS, and PCS [80, 90]. They enter the circulation through the impaired intestinal barrier and then exert harmful effects on the kidney. IS has the ability to promote the production of ROS in renal tubular epithelial cells; activate NF-*κ*B, p53, and other regulatory factors; and upregulate the expression of chemokines, leading to the aggregation of renal interstitial monocytes/macrophages and finally causing renal fibrosis [91]. IS can also promote the expression of transforming growth factor-*β* (TGF-*β*) and accelerate renal function deterioration by activating the renin-angiotensin-aldosterone system (RAAS) [92]. PCS has a proinflammatory effect, which can promote renal interstitial monocyte/macrophage infiltration and upregulate the expression of inflammatory factors, such as IL-6 and TGF-*β*, thus promoting renal fibrosis [93]. Both IS and PCS can lead to the hypermethylation of the Klotho gene, inhibit Klotho gene expression, weaken the protective effect of its products on the kidneys, and ultimately accelerate renal function deterioration [94]. In the circulation, 100% of the PCS and IS are bound to proteins, which limits their clearance; they could not be eliminated by dialysis. However, the binding capacity of proteins as a whole is decreased in patients with CKD [95], augmenting the circulating levels of unbound metabolites. The increased levels of PCS and IS in serum were positively correlated with renal degeneration, nephropathy progression, cardiovascular diseases, and mortality in patients with CKD [51]. Collectively, uremic toxins will trigger inflammation and tubulointerstitial damage and promote ROS production, tubular injury, and renal toxicity in the proximal renal tubular epithelial cells [96]. In addition to the tubules and mesenchymal damage, IS also injures the glomeruli where the podocytes play an important role. Podocytes are highly differentiated cells that are involved in the formation of glomerular filtration membranes and have a limited regenerative capacity [22]. Once the podocytes are damaged, proteinuria and other clinical manifestations of renal disease would occur. The abnormal increase of IS induces AhR activation, which contributes to the progressive impairment of the podocytes and glomeruli [97].

**Figure 3:**
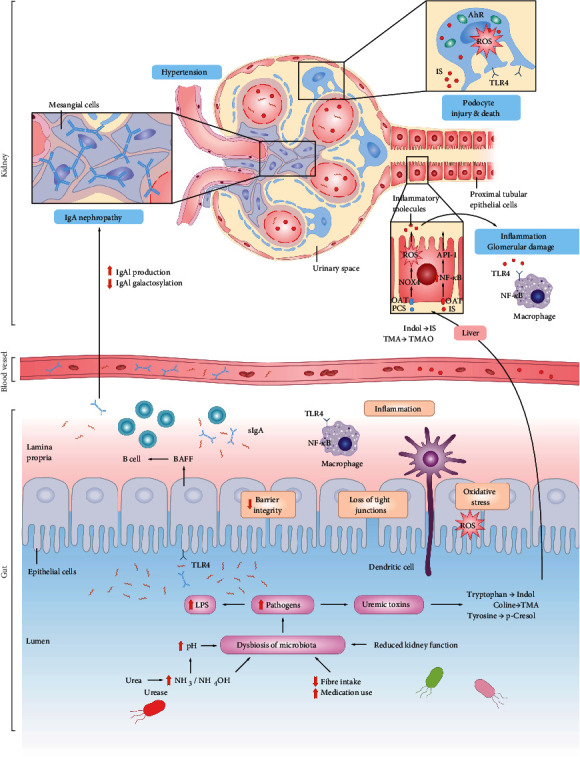
Changes in diet, numerous medication use, and decreased kidney function in CKD patients may lead to intestinal dysbiosis. The digestive tract becomes the main route for urea excretion in CKD patients with impaired renal function. A large amount of NH3 or NH4OH which are produced by bacteria can increase the intestinal pH value, promote the intestinal dysbiosis and destroy intestinal barrier. Thus, the transport of endotoxins (e.g., lipopolysaccharides (LPSs)) into the bloodstream has different effects on systemic inflammation, oxidative stress, cardiac injury, and atherosclerosis. Dysbiotic gut-derived uremic toxins, such as indoles and phenols, are further metabolized into trimethylamine N-oxide (TMAO), indoxyl sulfate (IS), and p-cresyl sulfate (PCS) in the liver and colon. The introduction of uremic toxins into the circulation causes inflammation and tubulointerstitial damage and promotes ROS production, tubulointerstitial damage, and nephrotoxicity of proximal tubuloepithelial cells. Intestinal microbial metabolites SCFAs are associated with hypertension and are important risk factors for CKD. SCFAs trigger hypertension through Olfr78, leading to renin secretion and regulation of peripheral resistance. In addition, bacteria and their components are involved in the hyperproduction and hypogalactosylation of IgA.

Renal disease is inextricably linked to cardiovascular diseases. Overall, 85%–90% of patients with CKD have hypertension [98], which is an important risk factor for CKD. Emerging studies have demonstrated a strong link between gut microbiota and hypertension in animals and patients [84, 99–103]. Yang et al. compared the fecal microbiome of spontaneously hypertensive rats and angiotensin-induced hypertensive rats. They noticed a prominent dysbiosis characterized by decreasing microbial abundance, variety, and evenness and the increased *Firmicutes*/*Bacteroidetes* ratio in hypertensive rats [103]. Moreover, treatments with antibiotics can lower the blood pressure of patients with treatment-resistant hypertension [102], indicating that the intestinal microbiome plays a role in hypertension pathogenesis and one of the possible causes is increased gut permeability and translocation of bacterial products [84]. Accumulating pieces of evidence suggest that gut-derived SCFAs contribute to the regulation of blood pressure via olfactory receptor 78 (Olfr78) and GPR41 [23, 84, 101, 104, 105]. Specifically, SCFAs trigger hypertension through Olfr78 in the peripheral blood vessels and renal afferent arterioles, leading to the secretion of renin and modulation of peripheral resistance when an intestinal microbial imbalance occurs. By contrast, SCFAs are able to lower blood pressure by binding with GPR41 and GPR43. Moreover, further research on the relationship between the intestinal microflora and the renal-cardiovascular system is helpful in the development of effective treatment methods for hypertension and CKD (Figure 3).

In view of the role of hypertension and other pathogenesis of CKD, the most useful management in the early stage of CKD is the control of blood pressure, along with reducing protein and salt intake to prevent acute renal injury and control blood glucose levels. With the exception of dialysis and kidney transplantation, no effective strategy to cure or prevent ESRD is currently available. Considering this, it could be hypothesized that regulating the intestinal microbiota can lower blood pressure, ameliorate kidney disease, and prevent complications in patients with CKD. Intervening measures (e.g., increasing fiber intake, rational use of antibiotics [106, 107], and therapeutic use of probiotics, prebiotics, and synbiotics) can restore the composition of intestinal flora and inhibit the accumulation of urotoxins in the blood [108, 109]. According to the research of Lakshmanan et al. [110], prebiotic gum acacia (GA) treatment restored the intestinal balance of CKD rats and relieves the inflammation of kidney tissue by increased production of butyrate, as well as its anti-inflammatory and antioxidant capacity. Future studies are needed to improve dialysis techniques to isolate protein-bound uremic toxins and to discuss the feasibility of fecal microflora transplantation.

## 4. Gut Microbiota in Diabetic Nephropathy

The worldwide prevalence of diabetes is rising rapidly, and it is estimated to increase to 578 million in 2030 [111]. Diabetes increases the risk of multiple complications, such as decreased kidney function and cardiovascular disease [112]. Although only 30%–40% of diabetic patients develop diabetic nephropathy (DN), it is a leading cause of ESRD in most developed countries and a key determinant of survival in people with diabetes [113]. Pathologically, the major changes in the kidney are the deposition of the extracellular matrix (ECM), thickening of the glomerular basement membrane, tubular atrophy, and cellular proliferation that results in interstitial fibrosis and glomerulosclerosis [113]. Accumulating pieces of evidence have revealed that increased ROS and low-grade inflammation, due in part to hyperglycemia, are strongly associated with diabetic complications [114, 115]. These changes lead to kidney damage, such as glomerular hyperfiltration, glomerular hypertension, altered glomerular composition, and hypernephrotrophy. Although the relationship is not clear, most studies believe that dysbiosis is involved in the occurrence and development of diabetes and DN, which may be related to the induction of insulin resistance and long-term chronic inflammation in diabetes.

Studies have investigated intestinal dysbiosis among diabetic patients and nondiabetic individuals [10, 11, 116], and it was found that intestinal dysbiosis is associated with insulin resistance and lipid metabolic disorders [117]. Intestinal dysbiosis itself and abnormal lipid metabolism in diabetes can decrease the expression of connective proteins, resulting in increased intestinal permeability and bacterial translocation. LPSs translocate into the circulation through the dysfunctional barrier and mediate host inflammatory responses through TLR2- and TLR4-related pathways, which is associated with the occurrence and development of many metabolic diseases. Chronic inflammation may also lead to the apoptosis of islet cells and eventually diabetes [7]. He et al. found that probiotics could delay the occurrence and development of diabetes by improving insulin resistance and stabilizing fasting blood glucose (FBG) levels [118]. The abovementioned studies have shown the correlation between intestinal flora and diabetes and found that restoring the gut microbiota is considered to be an effective strategy in preventing and treating diabetes.

In addition to the effect on insulin resistance, the intestinal microbiota may also be closely related to the occurrence and development of renal disease in diabetes through some other ways. Although magnesium lithospermate B is unable to decrease the FBG levels in STZ mice, the study of Zhao et al. showed that it can improve renal function (decreasing 24 h urinary protein) in diabetic patients by restoring the intestinal microbial composition and regulating the bile acid metabolism [119]. In 2019, one study first discovered a direct association between intestinal flora and DN. Through analyzing the fecal flora composition among diabetic biopsy-proven DN patients and healthy controls, the researchers found that the composition of the gut microbiota of DN patients is different from diabetic patients and healthy controls with several strains, such as *Escherichia*-*Shigella* [4]. The increased abundance of *Escherichia*-*Shigella* could penetrate the intestinal barrier and then exacerbate the intestinal leakage [120], which could contribute to the chronic low-grade inflammatory status in diabetic patients [121]. The interaction of bacterial LPS with TLR2 and TLR4 has been shown to be involved in the ongoing inflammatory process of DN by activating NF-*κ*B and inducing the release of proinflammatory cytokines (TNF, IL-1, IL-6, etc.) in an inflammatory cascade that exacerbates renal damage [118]. Moreover, the accumulation of toxic metabolites produced by intestinal microorganisms stimulates the production of ROS through the NADPH pathway, which triggers the NF-*κ*B pathway and induces an inflammatory response, and contributes to proteinuria and podocyte damage. Kikuchi et al. suggested that phenyl sulfate, a type of bacterial toxin, could potentially be an early diagnostic marker and a therapeutic target of DN in the future [122]. The NF-*κ*B pathway is a key point for the progression of inflammation and fibrosis in DN, whose activation can reduce the expression of inflammatory cytokines and fibrosis degree [123].

Recent studies have focused on the relationship between enterogenic products such as SCFAs and DN, which is a hot research field recently. Lu et al. speculated that intestinal microorganisms produce excessive SCFAs, especially acetate, which could bind to the renal Olfr78 receptor, and activate the intrarenal renin-angiotensin system (RAS) [124]. The activation of the RAS has long been regarded as one of the initiators of DN. The kidney is sensitive to angiotensin II (Ang II) which leads to renal vasoconstriction, increased blood pressure, and glomerular hypertension [125]. Moreover, Ang II promotes the morphological changes of podocytes and glomerular endothelial cells, the deposition of the extracellular matrix, and the secretion of inflammatory factors and profibrotic chemokines, accelerating the progress of DN. Furthermore, Hu et al. demonstrated that the acetate produced by the intestinal flora mediates the dysregulation of cholesterol homeostasis by activating GPR43, which leads to the tubulointerstitial injury of DN [126].

Clinical studies have demonstrated that traditional treatments that control glucose levels and inhibit RAS and inflammation could not absolutely prevent the progression of renal damage in DN. Considering this, the gut microbial factors could be involved in the pathogenesis of DN besides the traditional risk factors [127–129]. Modulating the gut microbiota may lead to better glycemic control and favorable outcomes in people with diabetes. Probiotics and prebiotics (e.g., fructooligosaccharides, lactulose, inulin, and resistant starches) are commonly used to regulate the gut flora, and the application of synbiotics and probiotics has been found to regulate the metabolic profile (e.g., glycemic, blood pressure, and lipid profile) of people with diabetes [130]. Studies have also reported the effect of probiotics and synbiotics in decreasing the biomarkers of inflammatory factors and oxidative stress [24, 131, 132], which could ameliorate kidney injury in diabetes. Chinese herbal medicine, QiDiTangShen granules, has been confirmed to modulate the gut microbiome composition and improved bile acid profiles in a mouse model of DN [133]. These benefits could be attributed to the ability of probiotics to restore epithelial barriers, producing SCFAs, modulating the immune response locally and systemically, and improving the gut barrier function. Moreover, in patients with type 2 diabetes, dietary fiber intake was strongly associated with glycemic control [11] and negatively associated with the prevalence of metabolic syndrome, both of which were associated with a lower risk of renal disease [134].

## 5. Gut Microbiota in Renal Ischemia-Reperfusion Injury

Renal ischemia-reperfusion injury (IRI) contributes to acute kidney injury (AKI) and delayed graft function after kidney transplantation [135]. Blood reperfusion of ischemic tissue increases the production of ROS that could attack the cells and tissue. Endogenous danger signals are released after cell stress and death, which could activate the tubules and endothelial cells to enhance the expression of adhesion molecules that can recruit innate and adaptive immune cells and promote ROS production [136]. Moreover, excessive ROS destroy the ratio of oxidant/antioxidant enzymes, leading to mitochondria-mediated cell apoptosis. Tubular epithelial cells and APCs secrete cytokines and chemokines that lead to the inflammatory response. APCs, such as macrophages and DCs, could activate CD4^+^ and CD8^+^ T lymphocytes by increasing the expression of total stimulus molecules, thereby leading to tissue damage [137].

According to the study of Emal et al., applying antibiotics leads to the diminution of the gut microbiome that can profoundly protect against kidney IRI by reducing the maturation status of the bone marrow monocytes and F4/80^+^ renal resident macrophages [138], suggesting that intestinal microbes play a role in the progression of ischemia-reperfusion injury to AKI. Furthermore, the treatment with the SCFAs (acetate, propionate, and butyrate) that the gut bacteria produced in the distal colon can improve renal dysfunction in mice with IRI. This protection was associated with the functions of SCFAs, such as reducing inflammation, cellular oxidative stress, and immune cell infiltration and regulate DNA methylation status [21].

## 6. Gut Microbiota in IgA Nephropathy

IgA nephropathy (IgAN) is the most common type of glomerulonephritis globally and a dominant cause of CKD and renal failure [139]. A characteristic of IgAN patients is the circulating elevation and glomerular accumulation of immune complexes consisting of aberrantly glycosylated IgA1, IgG autoantibodies, and C3, which leads to glomerular inflammation [139]. The generation of IgA in the intestinal mucosa is a predominant immunological process that is critical for homeostasis between the intestinal microbiota and the local immunological environment [140, 141]. Considering this, it could be hypothesized that gut dysbiosis and the abnormalities of the IgA mucosal immune system could be a significant element in the pathogenesis of IgAN [142].

The mucosal IgA is mainly produced in mesenteric lymph nodes (MLNs), Peyer's patches (PPs), and isolated lymphoid follicles (ILF) [140, 143]. The microenvironmental signals and controlling factors that drive the mass production of IgA in the intestine include the transforming growth factor-*β* (TGF-*β*) [144], BAFF, and APRIL [140, 145–147], which can reveal the commensal dependence in the IgA switch and the IgA-driven pathology [148]. Intestinal dysbiosis and chronic bacterial infections could stimulate the epithelial cells to produce BAFF and APRIL that promote the excessive production of IgA. Furthermore, studies have reported the distinct differences of the gut microbiome and metabolome in IgAN patients and healthy controls [149, 150] (Figure 3).

Otherwise, the potential link between the gut microbiota and the pathogenesis of IgAN could be revealed in the inhibition of IgA1 glycosylation by bacterial LPS. Qin et al. suggest that LPS could significantly inhibit the chaperone Cosmc, which is essential for the activity of galactosyltransferase, via toll-like receptor 4 (TLR4) [151]. The low Cosmc mRNA expression restrains the galactosylation level of IgA1 in IgAN patients. Combined with the fact that the bacterial LPS itself can stimulate a local and systemic inflammatory response, LPS is involved in the presence of the important features of IgAN pathogenesis including hyperproduction and hypogalactosylation of IgA1 [151].

## 7. Conclusion

The gut microbiome can be considered a giant bioreactor of the human body, which holds a bidirectional relationship with the host. Human-produced factors, such as sIgA and AMPs, can affect and control the intestinal microbiota potentially. The colonization of intestinal microorganisms is a double-edged sword for the host, and it can elicit a variety of effects on the host's health and diseases. A healthy microbial community plays an indispensable role in supporting symbiotic homeostasis by helping the body in resisting sudden changes from the internal and external environment, metabolizing nutrients, and secreting hormones, promoting the maturation of immune cells, maintaining the integrity of the intestinal epithelial barrier, and preventing the colonization of pathogenic microorganisms. A great deal of basic research can also confirm the role of microbiota in the treatment of a variety of renal disorders. The morbid state of the kidney leads to gut microbial dysbiosis, and in turn, gut microbial alteration induces renal injury. Imbalanced microbial composition leads to intestinal barrier permeability increase, accumulation of uremic toxins, and impaired autoimmune tolerance. Microbial dysbiosis and increased intestinal barrier permeability would be involved in the translocation of pathogenic bacteria, bacterial endotoxins, and toxic metabolites. Circulating microbial components may not lead to a clinical manifestation of infection but instead promote many pathological changes. Bacterial endotoxins and toxic metabolites cause chronic inflammation by activating the NF-*κ*B pathway and promoting the production of proinflammatory chemokines; bacterial pathogens destroy autoimmune tolerance and induce autoimmunity by causing an imbalance in the Treg/Th17 ratio and abnormal activation of B cells. Moreover, the alternation of the gut microbiota can increase oxidative stress and induce hypertension (Table 1). These processes are thought to contribute to the further progression of kidney diseases. Clinical evidence that clarified the intricate pathogenesis of kidney diseases from a gut microbial perspective has opened the possibility for the development of innovative treatments in copious microbial pathways as both potential pharmacological targets and mediators for renal diseases. It may be helpful in the long term to modulate the composition of intestinal flora and restore the epithelial barriers through diet, probiotics, and antibiotics. Ranganathan et al. [152] found that BUN levels showed statistically apparent differences in outcomes (*P* < 0.05) between the placebo and probiotic treatment periods at all four sites (46 patients). Oral administration of *Lactobacillus casei* or *L*. *acidophilus*, both of which can be used as probiotics, reduced the production of phenolic and indole uremic toxins significantly in hemodialysis patients [153]. In another set of trials, CKD patients who took *L*. *acidophilus* orally had a significant decline in their levels of serum urea concentration (dimethylamine and nitrosodimethylamine) [154]. In addition, a randomized controlled clinical trial demonstrated that synbiotic therapy significantly alters uremic toxin, PCS, and a palpable shift in the stool microbiome (particularly with the increase of *Bifidobacterium* and the decrease of *Ruminococcaceae*) [155]. Parasite-derived glycoprotein is involved in maintaining the balance of regulatory/effector B cells and desensitized renal effector function. Moreover, gut-derived SCFAs were proven to reduce inflammation, cellular oxidative stress, and immune cell infiltration and contribute to the regulation of DNA methylation status. These significant findings can contribute to the future development of treatment methods for renal diseases. Although numerous studies have been conducted, major advances are still needed to expand our understanding of the interaction mechanism between bacterial molecules and peripheral organs. Further investigations will be needed to prove the research achievement in animal models successfully in patients.

## Figures and Tables

**Table 1:** The related mechanism in the relationship between gut microbiota and kidney diseases.

Kidney diseases	Related mechanism	Conclusions	References

LN	Molecular mimicry	In susceptible individuals, symbiotic bacterial antigens cross-react with human DNA to activate the immune system and destroy self-tolerance, which is positively correlated with SLE activity and LN.	[70, 71]
	Treg/Th17 imbalance	Treg/Th17 imbalance can trigger immune responses and promote the production of SLE autoantibodies.	[40, 42]
	↑TLR7 and TLR9	An increase of TLR7 and TLR9 can contribute to alterations of proinflammatory cytokines in lupus patients.	[64, 65]
	Antinuclear antibodies	Mice with reduced gut bacteria developed nephritis more slowly and had lower levels of circulating antinuclear antibodies (ANAs) compared to the control group.	[67, 69]
		Germ-free lymphotoxin-deficient animals monocolonized with SFB produced more ANAs than lymphotoxin-deficient controls monocolonized with *E*. *coli*.	[68]

CKD	Endoxin	Endotoxemia can lead to systemic inflammation, oxidative stress, cardiac injury, and atherosclerosis.	[52]
	Uremic toxins (TMAO, IS, and PCS)	Uremic toxins cause inflammation and tubulointerstitial damage and promote ROS production, tubulointerstitial damage, epithelial cytotoxicity of proximal renal tubules, and progressive podocyte and glomerular damage.	[51, 96, 97]
	SCFAs	Gut-derived SCFAs trigger hypertension through Olfr78 in the peripheral blood vessels and renal afferent arterioles, which in turn leads to renin secretion and regulation of peripheral resistance.	[23, 84, 101]

DN	Insulin resistance	Intestinal dysbiosis is involved in insulin resistance and apoptosis of islet cells in diabetes.	[7, 10, 11, 116, 118]
	Activation of the RAS	Ang II accelerates the progression of DN by inducing renal vasoconstriction, promoting renal cell morphology, extracellular matrix deposition, inflammatory cytokine secretion, and fibro-promoting chemokines.	[125, 126]
	Uremic toxin	Phenyl sulfate can cause proteinuria and podocyte injury in diabetic mice. Inhibition of phenyl sulfate can reduce proteinuria in diabetic mice.	[122]

IRI	Bone marrow monocytes and renal resident macrophages	Applying antibiotics can diminish the gut microbiome and protect against kidney IRI profoundly by reducing the maturation status of bone marrow monocytes and F4/80^+^ renal resident macrophages.	[138]

IgAN	TGF-*β*, BAFF, and APRIL	Intestinal dysbiosis and chronic bacterial infections could stimulate epithelial cells to produce BAFF and APRIL which could promote excessive production of IgA.	[140, 144–147]
	Endoxin (LPS)	LPS is involved in the presence of important features of IgAN pathogenesis: hyperproduction and hypogalactosylation of IgA1.	[151]

## Abbreviations

SCFAs: Short-chain fatty acids
GPR41: G protein-coupled receptor 41
GPR43: G protein-coupled receptor 43
GPR109A: G protein-coupled receptor 109A
Treg cells: Regulatory T cells
NLRP3: NLR family pyrin domain containing 3
IL-18: Interleukin-18
HDACs: Histone deacetylases
IS: Indoxyl sulfate
PCS: p-Cresyl sulfate
TMAO: Trimethylamine N-oxide
AhR: Aryl receptor
AMPs: Antimicrobial peptides
M cells: Microfold cells
DCs: Dendritic cells
Th1: Helper T cell 1
Th2: Helper T cell 2
Th17: Helper T cell 17
IL-4: Interleukin-4
IL-5: Interleukin-5
IL-6: Interleukin-6
IFN-*γ*: Interferon-*γ*
APRIL: A proliferation-inducing ligand
BAFF: B cell-activating factor
sIgA: Secretory IgA
LN: Lupus nephritis
SLE: Systemic lupus erythematosus
TLRs: Toll-like receptors
TLR7: Toll-like receptor 7
TLR9: Toll-like receptor 9
IFN-*α*: Interferon-*α*
APCs: Antigen-presenting cells
C3a: Complement component 3a
C5: Complement component 5
IL-17A: Interleukin-17A
IL-17F: Interleukin-17F
CXCL5: C-X-C motif chemokine 5
CCL20: Chemokine (C-C motif) ligand 20
CCR6: Chemokine receptor 6
ROS: Reactive oxygen species
CKD: Chronic kidney disease
NF-*κ*B: Nuclear factor-kappa B
LPSs: Lipopolysaccharides
ESRD: End-stage renal disease
Olfr78: Olfactory receptor 78
DN: Diabetic nephropathy
ECM: Extracellular matrix
FBG: Fasting blood glucose
STZ: Streptozotocin
RAS: Renin-angiotensin system
Ang II: Angiotensin II
IRI: Ischemia-reperfusion injury
AKI: Acute kidney injury
IgAN: IgA nephropathy
MLNs: Mesenteric lymph nodes
PPs: Peyer's patches
ILF: Isolated lymphoid follicles
TGF-*β*: Transforming growth factor-*β*
TLR4: Toll-like receptor 4.
